# Investigation of Biomedical Students’ Attitudes toward Pharmacogenomics and Personalized Medicine: A Cross-Sectional Study

**DOI:** 10.3390/pharmacy10040073

**Published:** 2022-06-28

**Authors:** Josipa Bukic, Doris Rusic, Dario Leskur, Ana Seselja Perisin, Tin Cohadzic, Marko Kumric, Josko Bozic, Darko Modun

**Affiliations:** 1 Department of Pharmacy, University of Split School of Medicine, Soltanska 2, 21000 Split, Croatia; jbukic@mefst.hr (J.B.); drusic@mefst.hr (D.R.); dleskur@mefst.hr (D.L.); aperisin@mefst.hr (A.S.P.); 2 Department of Pediatric Surgery, University Hospital Centre Split, Spinciceva, 21000 Split, Croatia; tcohadzic@kbsplit.hr; 3 Department of Pathophysiology, University of Split School of Medicine, Soltanska 2, 21000 Split, Croatia; mkumric@mefst.hr (M.K.); jbozic@mefst.hr (J.B.)

**Keywords:** pharmacogenomics, education, attitudes, students, medicine, pharmacy

## Abstract

Background: The utilization of pharmacogenomics in everyday practice has shown several notable benefits. Keeping in mind the rising trend of applicability of pharmacogenomics and personalized medicine, we sought to compare the attitudes of future healthcare workers in different branches of the healthcare system. Methods: The present study was conducted as a questionnaire-based cross-sectional study in October of 2020. Students eligible to participate were all the students of the University of Split School of Medicine enrolled in the academic year 2020/2021. Results: The number of students that participated in the study was 503. Students were most interested in clinical examples of pharmacogenomics (31.4%) and the benefits of pharmacogenomics in clinical practice (36.4%). Furthermore, 72.6% of all students agreed that they should be able, in their future practice, to identify patients that could benefit from genetic testing. Conclusion: At the present time, the lack of education and appropriate clinical guidelines appear to be the major barriers to the clinical application of pharmacogenomics, especially in Croatia. Hence, in order to support health care professionals’ evidence-based therapeutic recommendations with patients’ pharmacogenomic data, universities should offer more pharmacogenomics education in their curricula.

## 1. Introduction

Utilization of pharmacogenomics, the science that studies how an individual’s genome could influence medication responses, into everyday practice has shown several notable benefits. Firstly, previous studies have described altered responses to certain medications in different patients, including specific medication doses, addressing the need for implementation of personalized, tailored dosing into clinical practice [1]. Recent advancements in terms of the use of health records and the rise in the number of available user-friendly software tools have resulted in the improvement of available clinical information and pharmacogenomics. In line with this, in cancer patients, therapeutic drug monitoring has added to the efficacy and safety of medication use whilst being cost-effective [2].

However, optimal integration of pharmacogenomics and personalized approach in clinical practice requires appropriate pharmacogenomics education of health care professionals, which was found to be lacking, especially in health care professionals who attended universities prior to the era of genomic medicine. In fact, the incorporation of pharmacogenomic education into academic settings has already been associated with several benefits. For instance, pharmacogenomic education improves the knowledge, interests and engagement of health care professionals, strengthens the overall genomic research and improves their communication skills about pharmacogenomics [3]. Therefore, the implementation of pharmacogenomic education at the university level has increased in the past few years [4]. The International Society of Pharmacogenomics has even proposed recommendations regarding pharmacogenomic education standards for the medical, pharmacy and health schools globally [5].

Previous studies showed positive attitudes of students towards pharmacogenomic education. For example, the results of Siamoglou et al.’s study revealed that students, despite their low level of awareness of pharmacogenomic testing, showed support for genetic testing and positive attitudes toward public endorsement of the pharmacogenomic concepts [6]. Moreover, the results of the study by Rahma et al. propose that the government should invest more money into pharmacogenomic education development. Furthermore, the authors interviewed academic staff and commissioners of the accreditation process at universities. The majority of participants stated that there is a need to recognize the importance of pharmacogenomics, and they called for the implementation of research as well as the creation of a standardized curriculum of pharmacogenomics in order to improve the education of pharmacogenomics and translation in the public health systems [7].

Keeping in mind the rising trend of applicability of pharmacogenomics and personalized medicine in everyday practice, we sought to compare the attitudes of future healthcare workers in different branches of the healthcare system. Hence, in this study, we compared attitudes between students of pharmacy, students of dental medicine, students of medicine and students of medicine in English, a group of students comprised mostly of foreign European students.

## 2. Materials and Methods

The present study was conducted as a questionnaire-based cross-sectional study in October of 2020. Students eligible to participate in our study were all the students of the University of Split School of Medicine enrolled in the academic year 2020/2021. The total number of students in the academic year 2020/2021 in pharmacy studies was 148, dental medicine studies 195, medical studies 669, and for medical studies in English, it was 289. The students’ participation was completely voluntary and anonymous. Furthermore, the study was approved by the Ethics Committee of the University of Split School of Medicine and was conducted in accordance with all the ethical principles of the Helsinki Declaration of 2013 [8].

Following the extensive literature review, we have chosen the questionnaire described in a study by Mahmutovic et al. for our research [9]. The questionnaire was first translated into Croatian language and then afterward back-translated into the English language by a native English speaker who did not participate in our study. The translated questionnaire was reviewed by 2 experts in the field of pharmacogenomics and personalized medicine for its clarity. Due to the pandemic of COVID-19, the questionnaire was distributed to students using the Google Form tool, a survey administration software offered by Google.

The questionnaire used in our study consisted five sections (Supplementary File S1). First, the questionnaire included the introductory glossary section in which students had available key definitions of pharmacogenomics and personalized medicine. The second section of the questionnaire, 7 items, included demographic data of the students. However, we added three questions in this section; country of origin (for medical studies in English only), study in which they are enrolled (for pharmacy, dental and medical students only) and question whether they have a family member working in a health-related field. The third section, 4 items, included information about students’ family anamnesis, health and experience with medications. The fourth section was comprised of 13 items about students’ attitudes toward genetic testing, personalized medicine and pharmacogenomics education. The last section included 11 items on ethical and social perspectives of pharmacogenomics, and we added two questions to this section. The first question examined whether students agreed national health care insurance should pay for the genetic testing, and the second question asked if students believed that genetic test results could influence students’ relationships with family/friends/partner. The questionnaire included yes/no/I do not know/and not sure answers, or the Likert scale (agree, disagree, no opinion or neutral) where applicable [9]. The results of our study are presented as whole numbers and proportions.

Statistical analysis was performed by using MedCalc (version 11.5.1.0, MedCalc Software, Ostend, Belgium). The categorical data were shown as absolute numbers (N) and percentages (%). The normality of data was assessed with the Kolmogorov–Smirnov test. Differences between groups of interest (different study programs) were assessed by using Chi-squared (χ2) test. Results with a *p*-value < 0.05 were considered statistically significant in all analyses.

## 3. Results

The number of all students that participated in the study was 503. Demographic data of students included in the study are presented in Table 1. The largest number of participants were pharmacy students (28.8%), followed by medical students (28.0%). Moreover, most of the included students were female, 75.1%, and were currently enrolled in the fifth year of study (21.7%). The largest proportion of students, 68.4%, had a grade point average of 4 (from 3.5 to 4.49).

Only a small number of students had a chronic heritable disease (15 students, 3.0%), and the majority of all students (231, 45.9%), without differences among different study programs, had family members that suffered from cancer. Almost 80% of students did not have any chronic disease and did not use medications regularly. Moreover, only 20.9% of all students experienced adverse drug reaction, and 24.2% of all students found out that a particular medication did not work for them during their lifetime. The majority of students (79.9%) think that genes moderately influence their health.

Interestingly, the largest proportion of all students, 58.2%, would consider having a genetic test done to find out what disease they might develop in the future. However, we observed a difference among different study programs, as proportion of pharmacy students who would get a genetic test was 55.9%, the proportion of dental students was 66.4%, the proportion of medical students was 49.6%, and proportion of students enrolled at the medical school in English was 63.6% (*p* = 0.009). Furthermore, majority of students (71.2%) heard about personal genome testing companies. Moreover, dissimilarities among students of different study programs were observed, as the proportion of pharmacy students that heard about companies was 76.5%, the proportion of dental students was 61.4%, the proportion of medical students was 68.0% and the proportion of English students was 84.4% (*p* = 0.002). However, only 24.8% of pharmacy students would consider contacting a personal genome testing company and order a pharmacogenomic test for themselves, while the percentage for dental students was 31.4%, 34.0% for medical students and 32.5% for medical students in English (*p* = 0.032).

A large proportion of all students, 80.9%, would be ready to make necessary lifestyle changes to reduce disease risk if they knew their genetic tendency to develop a certain disease. Moreover, 66.0% of all students would accept the pharmacogenomic test result and take a certain medication only if the disease might be life-threatening or if their pharmacogenomic test revealed that the prescribed medication would either be effective without severe side effects.

Students’ attitudes regarding personalized medicine and pharmacogenomics education are presented in Table 2.

Students were most interested in two pharmacogenomic topics, clinical examples of pharmacogenomics (31.4%) and benefits of pharmacogenomics in clinical practice (36.4%). Furthermore, 72.6% of all students agreed that they should be able, in their future practice, to identify patients that could benefit from genetic testing, and 70.0% agreed that they should be able to identify drugs that would require pharmacogenomics testing prior to their administration. The majority of students, 71.2%, were aware of different ethical aspects of genetic testing. Moreover, students addressed data confidentiality as the most common ethical issue related to genetic or pharmacogenomic testing (28.8%).

The largest proportion of students, 71.4%, indicated physicians as healthcare professionals that should have access to pharmacogenomic information, followed by pharmacists (11.9%) and genetic counselors (11.3%). A little over half of all the students (56.5%) stated they are slightly worried about the possibility that a pharmacogenomic test may reveal additional risk factors for other diseases. Thirty-eight percent of all students believed that they would be disadvantaged at work or job-seeking in case an unfavorable test result should be disclosed. Moreover, among different study programs, students of medical studies in English accounted for the largest proportion of students that believed in the aforementioned disadvantage, and dental students accounted for the smallest proportion. Furthermore, dental students least commonly believed that they would feel “helpless” or “pessimistic” in case of an unfavorable test result, in comparison to pharmacy, medical and English students.

Half of the students, 51.3%, believed that in the future, pressure may be exerted on patients to agree to perform a pharmacogenomic test. However, the difference among students in four study programs was observed (*p* = 0.012), and proportion of students with the aforementioned belief was 56.6%, 38.6%, 52.5% and 62.3% for pharmacy, dental, medical and English students, respectively. Similarly, the majority of students (58.6%) agreed that national health care insurance should pay for the genetic testing with proportions of 63.2%, 57.9%, 46.8% and 72.7% for pharmacy, dental, medical and English students, respectively, *p* = 0.003. Furthermore, 49.1% of students believed that test results could influence their relationship with family/friends/partner, with proportions of 45.5%, 38.6%, 52.5% and 69.3% for pharmacy, dental, medical and English students, respectively, *p* < 0.001.

## 4. Discussion

The results of the present study have shown that of the included students (84.3%), most notably pharmacy students, agreed that personalized medicine presents a promising healthcare model. However, 58.2% of all students would consider having a genetic test performed, and only 18.5% would continue postgraduate education in the field of personalized medicine. Furthermore, even though majority of students think that pharmacogenomics should be an important part of the study curriculum, less than 12% believe that the curriculum is adequately designed to understand pharmacogenomics. Among pharmacogenomic topics, students were most interested in clinical examples of pharmacogenomics and the benefits of pharmacogenomics in clinical practice, as each of these topics was selected by approximately one-third of students. Finally, among ethical concerns, half of the students addressed the issue of pressure that may be exerted on patients to agree to perform a pharmacogenomic test. Nevertheless, among different studies, students had different beliefs with regard to this issue also in terms of financing these testing. In addition, half of the students believe that test results could influence their relationship with family/friends/partners. To the best of our knowledge, this is the first study that analyzed the awareness and attitudes towards pharmacogenomics and personalized medicine among students of biomedical studies in Croatia.

Our results indicate that although most of the students are aware of the importance that personalized medicine will have in future clinical practice, most of the students are not interested in postgraduate education in this field. These results are in concordance with the available data, which indicates that healthcare students believe that pharmacogenomics is important for patient care [10] and that they should have the knowledge to perform genetic test results in order to optimize therapeutic modalities and educate their patients [11]. However, our results are different from those obtained by Mahmutovic et al., since in their study, only 57% of all students and 70% of pharmacy students answered affirmatively to this question [9]. The observed discrepancy likely reflects the regional differences in curricula, as based on our comparison, personalized medicine is much more integrated into our university. Conversely, a much larger proportion of students from Bosnia (53%) would consider continuing postgraduate education in the field of personalized medicine. Moreover, Vaksman et al. reported that the majority of students from the pharmacy schools in California were aware of pharmacogenomics and its importance for the future pharmacist and would be interested in a residency and/or career specializing in this field. Nevertheless, the same authors demonstrated that the presence of a stand-alone pharmacogenomics course did not impact student-perceived preparedness for a career in that field [12]. Interestingly, in the present study, dental medicine students had the least belief with regard to the role of personalized medicine in their future clinical practice. These results are hard to interpret since there are no data to compare, but we can hypothesize that these results arise as a consequence of the low enrolment rate in courses that cover the topic of personalized medicine among our dental medicine students.

Surprisingly, only 58% of our students would consider having a genetic test done to find out what illnesses they might develop in the future, and only about one-fourth of pharmacy students would consider contacting a personal genome testing company and ordering a pharmacogenomic test for themselves. Among different courses, medical students were most prone to ordering a pharmacogenomic test for themselves. In a study by Siamoglou et al., in which attitudes were assessed in pharmacy and medicine students in Malaysia, 80% of pharmacy and medical students wanted to have a genetic test done, indicating greater awareness in comparison to our students [6]. Accordingly, Rahma et al. demonstrated on students enrolled in medicine and health sciences in the United Arab Emirates that 82.7% would consider performing a genetic test [13]. Rahma et al. further explored this issue in various settings and found that most students would have conducted the test only if intervention for prevention of the disease development was available, and if, provided that the patient had a cancer diagnosis, the test could help in the prevention of cancer for other family members. In line with this, a study by Adams et al. even proposed personal genomic testing to enhance pharmacogenomics education [14]. The authors conveyed that majority of included students reported a better understanding of pharmacogenomics due to the personal testing and real genetic data. 

Only 12% of pharmacy students in our study agreed that their curriculum is well designed to grasp pharmacogenomics. On the other hand, 50% of both Bosnian students and pharmacy students at the University of Minnesota claimed that their curriculum is appropriately designed to understand and apply pharmacogenomics [15]. Similarly, the results of the study by Jarrar et al. showed that pharmacy students in Jordan and Palestine wished to know more about pharmacogenomics, and 60.3% of students stated their pharmacogenomics education was insufficient [16]. Poor approval of the current curriculum with respect to pharmacogenomics strongly addresses the need for a change of the current curriculum in our University. Two studies reported that pharmacogenomics education in medical and pharmacy studies is most frequently offered as part of the pharmacology curricula, followed by elective and mandatory courses, yet more importantly, the studies showed that half or more universities that did not offer pharmacogenomic education did not plan to implement it in the near future [17]. This is troublesome, as multiple data suggest beneficial effects of the implementation of pharmacogenomic education. Specifically, McCullough et al. demonstrated that education emphasizing clinical applications of pharmacogenomics can significantly increase students’ knowledge and comfort in their practice [10]. 

Furthermore, a systematic review by Talwar et al. summarized the characteristics and evaluation outcomes of pharmacogenomic curricula offered to health care professional students [18]. The review included 41 studies, the majority of which were conducted in the United States, followed by the United Kingdom, Canada, the Netherlands and China. The review results showed that incorporation of pharmacogenomics in the curricula had positive effects on students’ knowledge, attitudes, self-efficacy, comfort level and motivation. However, 68.3% of the included studies did not have theory-based genomics curricula, and 85.4% of the studies did not report data of the follow-up. Accordingly, in a recent study, Pisanu et al. assessed the discrepancy in pharmacogenomic education in Southeast Europe and recommended that this subject should be thought of as a stand-alone course or at least as a part of the existing courses in genetics [19]. Of important note, in a study conducted at the Stanford School of Medicine, the authors demonstrated that almost all students taking a course in personalized medicine believed that physicians are not trained to interpret the results of pharmacogenomic tests and are thus not capable of practicing personalized medicine [20]. Novel data suggest that the use of active learning experiences in pharmacogenomics can substantially increase student interest in the topic [21]. In addition, recently adopted innovative learning modalities such as Pharmacogenomics Education Program 3 online courses and personal genotyping could in the near future teach students to use genetic information in the framework of medication management, allowing them to understand and utilize pharmacogenomic knowledge in clinical practice.

The lack of education and appropriate clinical guidelines appear to be the major barriers to the clinical application of pharmacogenomics, as perceived by participants [9]. This education can be offered through continuing education of health care professionals or as formal education of health care students. In order to support health care professionals’ evidence-based therapeutic recommendations with patients’ pharmacogenomic data, universities should offer an adequate amount of pharmacogenomics education in their curricula. As pharmacogenomics has been expanded in the past few decades into more areas of health care, and as it is becoming one of the most relevant aspects of patient care, health care professionals must pursue and maintain competences in this field in order to ensure an improved outcome for their patients [22]. In line with this, pharmacogenomics makes a more accurate method of determining appropriate drug dosages and allows a patient to make adequate lifestyle changes in order to avoid genetic diseases. Therefore, it is essential that health care professionals are offered continuing education on the matter of pharmacogenomics, as this education would accelerate the clinical implementation of pharmacogenomic services [23].

The fact that personalized medicine and pharmacogenomics bear a substantial ethical burden has been recognized by most of our students. In the present study, students addressed data confidentiality as the most common ethical issue related to genetic or pharmacogenomic testing. The largest proportion of students indicated physicians as healthcare professionals that should have access to pharmacogenomic information, followed by pharmacists and genetic counselors. Only a small proportion of participants included in our study identified genetic counselors as healthcare professionals that should have access to pharmacogenomic information. However, it should be acknowledged that there is no specific education for the profession of genetic counselor and that these professionals are not widely available at Croatian hospitals. Notwithstanding, there is a possibility that there will be an increase in educational paths and possibilities for the professional advancement of genetic counselors in the future [24]. More than a third of all students believed that they would be disadvantaged at work or job-seeking in case an unfavorable test result should be disclosed. These results are in line with those obtained by Mahmutovic et al. [9]. Moreover, among different study programs, students of medical studies in English accounted for the largest proportion of students that believed in the aforementioned disadvantage and were most afraid that test results could influence their relationship with family/friends/partner. The observed difference probably reflects the difference between the ethical standpoints of students from less developed parts of Europe (Bosnia and Croatia) and Western Europe, as most of our students originate from that area. Interestingly, in a study by Rahma et al., as many as two-thirds of students believe that pharmacogenomics results may be exploited by employers and insurance companies [13]. Finally, in accordance with the available data, which indicated that each individual would respond differently to the genetic test results, around half of our students believed that they would feel neither “helpless”/“pessimistic” nor “different”/“inadequate” in case of an unfavorable test result [24]. Notably, dental students were the least concerned with the above-noted issue in comparison to pharmacy, medical and English students.

The present study bears several notable limitations. Firstly, the diverse ethnicity of foreign students leads to dispersion of data, thus preventing nation-specific analysis. Furthermore, the study was performed in only one center (University of Split). Furthermore, our study assessed perceived (or self-reported) understanding and skills in pharmacogenomics and personalized medicine without evaluating the actual students’ knowledge and capabilities. Notably, survey tools that use the Likert scale, such as the one we employed, are prone to central tendency bias due to the selection of neutral answers. Finally, we did not investigate which teaching tools students would favor in determining the most effective way for education in terms of pharmacogenomics and personalized medicine. Nevertheless, despite these limitations, our study provides an important reference point for future comparative studies between different regions and different arms of the healthcare system.

## 5. Conclusions

As pharmacogenomics is becoming one of the most relevant aspects of patient care, health care professionals must pursue and maintain competencies in this field in order to ensure an improved outcome for their patients. Therefore, it is essential that health care professionals are offered continuing education on the matter of pharmacogenomics, as this education would accelerate the clinical implementation of pharmacogenomic services. At the present time, the lack of education and appropriate clinical guidelines appears to be a major barrier to the clinical application of pharmacogenomics, especially in our area. Hence, in order to support health care professionals’ evidence-based therapeutic recommendations with patients’ pharmacogenomic data, universities should offer more pharmacogenomics education in their curricula. Nevertheless, in order to adequately implement pharmacogenomics and a personalized approach in practice, ethical concerns burdening the implementation of this concept should also be carefully resolved and legislated.

## Figures and Tables

**Table 1:** Demographic data of students.

	Pharmacy N (%)	Dental Medicine N (%)	Medicine N (%)	Medical Studies in English N (%)	Total N (%)	*p*-Value *
Number of participants	145 (28.8)	140 (27.8)	141 (28.0)	77 (15.4)	503 (100)	<0.001
Gender						
Female	126 (86.9)	111 (79.3)	93 (66.0)	48 (62.3)	378 (75.1)	<0.001
Male	19 (13.1)	28 (20.7)	48 (34.0)	29 (37.7)	124 (24.9)	
Study year						
1	18 (15.4)	0	0	17 (22.1)	35 (7.0)	<0.001
2	30 (20.7)	37 (26.4)	3 (2.1)	33 (42.9)	103 (20.5)	
3	27 (18.6)	32 (22.9)	20 (14.2)	8 (10.4)	87 (17.3)	
4	26 (17.9)	34 (24.3)	23 (16.3)	4 (5.2)	87 (17.3)	
5	44 (27.4)	11 (7.9)	44 (31.2)	10 (13.0)	109 (21.7)	
6	n/a	26 (18.5)	51 (36.2)	5 (6.4)	82 (16.2)	
GPA						
2	0	0	1 (0.7)	1 (1.3)	2 (0.4)	<0.001
3	18 (12.4)	42 (30.0)	13 (9.2)	25 (32.5)	98 (19.5)	
4	104 (74.3)	85 (60.7)	110 (78.0)	45 (58.4)	344 (68.4)	
5	23 (13.3)	13 (9.3)	17 (12.1)	6 (7.8)	59 (11.7)	

* Chi-square test. n/a—not applicable; GPA—grade point average.

**Table 2:** Students’ attitudes about personalized medicine and pharmacogenomic education.

	Pharmacy N (%)	Dental medicine N (%)	Medicine N (%)	Medical studies in English N (%)	Total N (%)	*p*-Value *
Personalized medicine presents promising healthcare model						
Yes	137 (94.5)	98 (70.0)	127 (90.1)	62 (80.5)	424 (84.3)	*p* < 0.001
No	1 (0.7)	6 (4.3)	2 (1.4)	2 (2.6)	11 (2.2)	
Do not know	7 (4.8)	36 (25.7)	12 (8.5)	13 (16.9)	68 (13.5)	
Pharmacogenomics should be an important part of study curriculum						
Agree	117 (80.7)	62 (44.3)	70 (49.6)	37 (48.1)	286 (56.8)	*p* < 0.001
Disagree	4 (2.7)	18 (12.8)	18 (12.8)	11 (14.3)	51 (10.1)	
No opinion	24 (16.6)	60 (42.9)	53 (37.6)	29 (37.6)	166 (33.1)	
Curriculum is well designed to understand pharmacogenomics						
Yes	18 (12.4)	9 (6.4)	25 (17.7)	8 (10.4)	60 (11.9)	*p* < 0.001
No	35 (24.1)	66 (47.1)	57 (40.4)	18 (23.4)	176 (35.0)	
Do not know	41 (28.3)	28 (20.0)	24 (17.0)	33 (42.9)	126 (25.0)	
Not sure	51 (35.2)	37 (26.5)	35 (24.9)	18 (23.3)	141 (28.0)	
Continue postgraduate education in the field of personalized medicine						
Yes	26 (17.9)	33 (23.6)	22 (15.6)	12 (15.6)	93 (18.5)	*p* < 0.001
No	30 (20.7)	59 (42.1)	53 (37.6)	36 (46.8)	178 (35.4)	
I do not know	36 (24.8)	27 (19.3)	44 (31.2)	17 (22.1)	124 (24.7)	
Not sure	53 (36.6)	21 (15.0)	22 (15.6)	12 (15.6)	108 (21.5)	

* Chi-square test.

## Data Availability

The datasets generated and analyzed during the current study are available from the corresponding author on reasonable request.

## Supplementary Materials

The following supporting information can be downloaded at: https://www.mdpi.com/article/10.3390/pharmacy10040073/s1, Supplementary File S1: Survey questionnaire.

## References

[1] Anesh T.P., Sonal S., Jose A., Chandran L., Zachariah S.M. (2009). Pharmacogenomics: The right drug to the right person. J. Clin. Med. Res..

[2] Vithanachchi D.T., Maujean A., Downes M.J., Scuffham P. (2021). A comprehensive review of economic evaluations of therapeutic drug monitoring interventions for cancer treatments. Br. J. Clin. Pharmacol..

[3] Rohrer Vitek C.R., Abul-Husn N.S., Connolly J.J., Hartzler A.L., Kitchner T., Peterson J.F., Rasmussen L.V., Smith M.E., Stallings S., Williams M.S. (2017). Healthcare provider education to support integration of phar, macogenomics in practice: The eMERGE Network experience. Pharmacogenomics.

[4] Whitley K.V., Tueller J.A., Weber K.S. (2020). Genomics Education in the Era of Personal Genomics: Academic, Professional, and Public Considerations. Int. J. Mol. Sci..

[5] Gurwitz D., Lunshof J.E., Dedoussis G., Flordellis C.S., Fuhr U., Kirchheiner J., Licinio J., Llerena A., Manolopoulos V.G., Sheffield L.J. (2005). Pharmacogenomics education: International Society of Pharmacogenomics recommendations for medical, pharmaceutical, and health schools deans of education. Pharm. J..

[6] Siamoglou S., Koromina M., Politopoulou K., Samiou C.G., Papadopoulou G., Balasopoulou A., Kanavos A., Mitropoulou C., Patrinos G.P., Vasileiou K. (2021). Attitudes and Awareness Toward Pharmacogenomics and Personalized Medicine Adoption Among Health Sciences Trainees: Experience from Greece and Lessons for Europe. Omics A J. Integr. Biol..

[7] Rahma A.T., Ahmed L.A., Elsheik M., Elbarazi I., Ali B.R., Patrinos G.P., Al-Maskari F. (2021). Mapping the Educational Environment of Genomics and Pharmacogenomics in the United Arab Emirates: A Mixed-Methods Triangulated Design. OMICS J. Integr. Biol..

[8] World Medical Association (2013). World Medical Association Declaration of Helsinki: Ethical principles for medical research involving human subjects. JAMA.

[9] Mahmutovic L., Akcesme B., Durakovic C., Akcesme F.B., Maric A., Adilovic M., Hamad N., Wjst M., Feeney O., Semiz S. (2018). Perceptions of students in health and molecular life sciences regarding pharmacogenomics and personalized medicine. Hum. Genom..

[10] McCullough K.B., Formea C.M., Berg K.D., Burzynski J.A., Cunningham J.L., Ou N.N., Rudis M.I., Stollings J.L., Nicholson W.T. (2011). Assessment of the pharmacogenomics educational needs of pharmacists. Am. J. Pharm. Educ..

[11] Moen M., Lamba J. (2012). Assessment of healthcare students’ views on pharmacogenomics at the University of Minnesota. Pharmacogenomics.

[12] Vaksman N., Barnett M., Hakobyan L., Kutcher I., Louie M.C. (2012). The Impact of Incorporating of Pharmacogenomics into the Pharmacy Curriculum on Student Interest. Pharm. Educ..

[13] Rahma A.T., Elsheik M., Elbarazi I., Ali B.R., Patrinos G.P., Kazim M.A., Alfalasi S.S., Ahmed L.A., Al Maskari F. (2020). Knowledge and Attitudes of Medical and Health Science Students in the United Arab Emirates toward Genomic Medicine and Pharmacogenomics: A Cross-Sectional Study. J. Pers. Med..

[14] Adams S.M., Anderson K.B., Coons J.C., Smith R.B., Meyer S.M., Parker L.S., Empey P.E. (2016). Advancing Pharmacogenomics Education in the Core PharmD Curriculum through Student Personal Genomic Testing. Am. J. Pharm. Educ..

[15] Marcinak R., Paris M., Kinney S.R.M. (2018). Pharmacogenomics Education Improves Pharmacy Student Perceptions of their Abilities and Roles in Its Use. Am. J. Pharm. Educ..

[16] Jarrar Y., Mosleh R., Hawash M., Jarrar Q. (2019). Knowledge and Attitudes Of Pharmacy Students Towards Pharmacogenomics Among Universities in Jordan And West Bank Of Palestine. Pharm. Pers. Med..

[17] Karas Kuzelicki N., Prodan Zitnik I., Gurwitz D., Llerena A., Cascorbi I., Siest S., Simmaco M., Ansari M., Pazzagli M., Di Resta C. (2019). Pharmacogenomics education in medical and pharmacy schools: Conclusions of a global survey. Pharmacogenomics.

[18] Talwar D., Chen W.J., Yeh Y.L., Foster M., Al-Shagrawi S., Chen L.S. (2019). Characteristics and evaluation outcomes of genomics curricula for health professional students: A systematic literature review. Genet. Med..

[19] Pisanu C., Tsermpini E.E., Mavroidi E., Katsila T., Patrinos G.P., Squassina A. (2014). Assessment of the pharmacogenomics educational environment in Southeast Europe. Public Health Genom..

[20] Salari K., Karczewski K.J., Hudgins L., Ormond K.E. (2013). Evidence that personal genome testing enhances student learning in a course on genomics and personalized medicine. PLoS ONE.

[21] Knoell D.L., Johnston J.S., Bao S., Kelley K.A. (2009). A genotyping exercise for pharmacogenetics in pharmacy practice. Am. J. Pharm. Educ..

[22] Guy J.W., Patel I., Oestreich J.H. (2020). Clinical Application and Educational Training for Pharmacogenomics. Pharmacy.

[23] Baty B.J. (2018). Genetic counseling: Growth of the profession and the professional. Am. J. Med. Genet. Part C Semin. Med. Genet..

[24] Howard H.C., Borry P. (2013). Survey of European clinical geneticists on awareness, experiences and attitudes towards direct-to-consumer genetic testing. Genome Med..

