# Supplementary material for: Investigation of Biomedical Students’ Attitudes toward Pharmacogenomics and Personalized Medicine: A Cross-Sectional Study
Source: Pharmacy (Basel). 2022 Jun 28;10(4):73. doi: 10.3390/pharmacy10040073 (PMC9326582; doi:10.3390/pharmacy10040073)
Supplement: Supplementary file 1 [file pharmacy-10-00073-s001.zip › pharmacy-1768298-supplementary.pdf]

This survey is anonymous and will be used for research purposes only. It will take approximately 15 minutes of your time. Please, answer all questions as honestly as you can. All your answers will be completely confidential. If for any reason, you do not feel comfortable answering any specific question, you can decline to answer it.

Key definitions:

“Genetic testing" analyzes an individual’s genetic material that will help patients and their doctors to identify a person with a predisposition for a particular disease; detect whether a person has a disease; identify the effectiveness and potential risk of side effects of a particular drug for an individual patient.

"Personalized medicine" refers to an innovative approach to disease diagnosis and treatment that takes into account differences in people's genes, environment, and lifestyles.

“Pharmacogenomics“ is the study of the association of an individual’s genetic variation with drug response.

“Pharmacogenomic test” analyzes an individual’s genetic material (DNA), in order to find out if they will benefit from a drug, require a different dose, or experience side effects. For example, the CYP450 test identifies variation in two genes, CYP2D6 and CYP2C19, which are associated with the metabolism of at least 50 commonly prescribed drugs. Variation in CYP2D6 gene can result in low/slow/poor, normal/rapid/extensive, or ultra-rapid metabolism of some drugs. Those patients who metabolize the drug slowly are at increased risk of having the drug remain in their blood for a prolonged period, which in turn increases their risk of adverse side effects. Ultra-rapid metabolizers may not achieve sufficiently high levels in their blood to have therapeutic effect since the drug is metabolized and eliminated too quickly.

By completing this survey, I am giving my informed consent to participate in this research.

Survey Questionnaire

1. Country of origin _____________

2. GPA
   2 3 4 5

3. Family member working in healthcare system
   Yes No

4. Year of study
   1 2 3 4 5 6

5. Mark your gender
   Male Female

6. Mark which is correct about you
   a) I have a chronic heritable disease
   b) I use pharmacotherapy everyday
   c) My family members had cancer
   d) My family members had heritable disease

7. What is your family average monthly income?
   a) <2000 HRK
   b) 2000-4000
   c) 4000-8000
   d) >8000 HRK
   e) do not want to respond

8. Have you been diagnosed with any of following diseases? You can choose multiple options.
   a) Cardiovascular (heart problems, atherosclerosis,
   b) Psychiatry (depression, anxiety)
   c) Metabolic diseases (diabetes, metabolic syndrome)
   d) Oncology
   e) Other ________
   f) No

9. Did you ever take a drug that is used to treat any of the following diseases? You can choose multiple options.
   a) Cardiovascular (heart problems, atherosclerosis,
   b) Psychiatry (depression, anxiety)
   c) Metabolic diseases (diabetes, metabolic syndrome)
   d) Oncology

e) Other ________
f) No

10. Have you ever had an adverse drug reaction?
   a) Yes
   b) No
   c) Don't know
   d) I have never taken any medication

11. Have you ever found that a particular drug did not work for you?
   a) Yes
   b) No
   c) Don't know
   d) I have never taken any medication

12. To what extent do you think that genes influence your health?
   a) Completely
   b) Not at all
   c) Moderately
   d) Don't know

13. Would you consider having a genetic test done to find out what illnesses you might develop in the future?
   a) Yes
   b) No
   c) Don't know

14. Have you heard about personal genome testing companies?
   a) Yes
   b) No
   c) Don't know
   d) Not sure

15. Would you consider contacting personal genome testing company and ordering a pharmacogenomic test for yourself?
   a) Yes
   b) No
   c) Don't know
   d) Not sure

16. If you know your genetic tendency to develop a disease, would you be ready to make necessary changes in your lifestyle, to reduce disease risk?
   a) Yes
   b) No

c) Maybe
d) Don't know

17. If a pharmacogenomic test revealed that prescribed drug would either be ineffective or cause severe side effects, would you take the drug anyway?
   a) Take the drug anyway
   b) Accept the test result, and not take the drug
   c) Accept the test result, and take the drug only if the disease might be life-threatening
   d) Not sure

18. Do you agree that personalized medicine represent a new and promising healthcare model?
   a) Yes
   b) No
   c) Don't know

19. Pharmacogenomics should be an important part of my study curriculum.
   a) Agree
   b) Disagree
   c) Neutral
   d) No opinion

20. Do you think that curriculum of your study program is well designed for understanding pharmacogenomics?
   a) Yes
   b) No
   c) Don't know
   d) Not sure

21. In my future practice, I should be able to identify patients that could benefit from genetic testing.
   a) Agree
   b) Disagree
   c) Neutral
   d) No opinion

22. In my future practice, I should be able to answer patient's questions regarding pharmacogenomics and personalized medicine?
   a) Agree
   b) Disagree
   c) Neutral
   d) No opinion

23. In my future practice, I should be able to identify drugs that would require pharmacogenomics testing prior to their administration to the patient.
  a) Agree
  b) Disagree
  c) Neutral
  d) No opinion

24. Would you like to continue your postgraduate education (master, PhD, specialization) in the field of personalized medicine?
  a) Yes
  b) No
  c) Don't know
  d) Not sure

25. Which pharmacogenomics topics would you be interested to learn more about? You can choose multiple options.
  a) Pharmacogenomics in general
  b) Benefits of pharmacogenomics in pharmacogenomics
  c) Future development in pharmacogenomics
  d) Clinical examples of clinical practices
  e) Ethical, legal and social issues
  f) Other ___________________

26. How much money are you willing to spend to examine the effectiveness of a specific drug in your body using pharmacogenomic test?
  a) <600 HRK
  b) 600-2000 HRK
  c) 2000-4000 HRK
  d) >4000 HRK
  e) Not sure
  f) I would not pay for testing

27. Are you aware of different ethical aspects of genetic testing?
  a) Yes
  b) No
  c) Not sure

28. What ethical issues do you believe might be related to genetic or pharmacogenomic testing?
  a) Patient privacy
  b) Racial issues
  c) Non-incidental findings
  d) Data confidentiality
  e) Stigma

f) Other

29. Are you worried about the possibility that the result of a pharmacogenomic test may be passed to unauthorized persons?
a) Very worried
b) Not worried
c) Slightly worried
d) I don't know

30. Which of the following healthcare professionals should have access to your pharmacogenomic information? You can choose multiple options.
a) Physician
b) Genetic counselor
c) Psychologist
d) Pharmacist
e) Social worker
f) Nurse
g) Nutritionist

31. Are you worried about the possibility that a pharmacogenomic test may reveal that you have additional risk factors for other diseases?
a) Very worried
b) Not worried
c) Slightly worried
d) No opinion

32. In case an unfavorable test result should be disclosed, do you believe that you would be disadvantaged at work or job-seeking?
a) Yes
b) No
c) No opinion

33. In case of an unfavorable test result, do you believe that you would feel “helpless” or “pessimistic”?
a) Yes
b) No
c) No opinion

34. Do you believe that in the future pressure may be exerted on patients to agree to perform a pharmacogenomic test?
a) Yes
b) No
c) No opinion

35. Do you agree that national health care insurance should pay for genetic testing?

a) Yes

b) No

c) No opinion
